# Medical students' distress during the transition to the endemic phase of COVID-19 in China: The association with temperament traits and attachment styles

**DOI:** 10.3934/publichealth.2025001

**Published:** 2025-01-03

**Authors:** Carmenrita Infortuna, Xiaolin Yang, Ray Wang, Gianluca Pandolfo, Ilona Cazorla, Julian Dupont, Veolette Hanna, Valerie Iosim, Mirai Mikhail, Alex Yu, Stanley R. Terlecky, Florian P. Thomas, Jing Ren, Wenhua Cao, Zhiyong Han, Fortunato Battaglia

**Affiliations:** 1 Department of Biomedical and Dental Sciences and Morphofunctional Imaging, Policlinico Universitario, University of Messina, Messina, Italy; 2 Research Center of Narrative Medicine, Shunde Hospital; Department of General Education, Southern Medical University, Guangdong, China; 3 Williams College, Williamstown, MA, USA; 4 Department of Medical Sciences, Hackensack Meridian School of Medicine, Nutley, NJ, USA; 5 Department of Neurology, Hackensack Meridian School of Medicine, Nutley, NJ, USA; 6 The Second School of Clinical Medicine, Southern Medical University, Guangzhou, China; 7 Department of Psychology, Hunan Normal University, Changsha, China

**Keywords:** temperament, attachment styles, endemic, medical students, stress

## Abstract

**Objective:**

The transition of COVID-19 into the endemic phase in China has posed additional challenges to medical student's well-being, and increased the odds of mental distress. Although affective temperament traits and adult attachment styles accompany crisis-induced stress, whether this applies to medical students in the endemic phase has yet to be determined. The aim of present study is to test if temperament traits and adult attachment style can predict stress in Chinese medical students.

**Methods:**

Medical students (*N* = 402) enrolled in the undergraduate medical program at the Southern Medical University, Guangdong, China completed an online survey in May 2022. Most participants were female (62.4%), with a mean age of (21.3 ± 3.1). The individual temperament traits and adult attachment styles were assessed using the Chinese version of the Temperament Evaluation of Memphis, Pisa, Paris, and San Diego-auto-questionnaire short version (TEMPS-A), and the Attachment Style Questionnaire (ASQ).

**Results:**

The participants showed significant distress as assessed with the K10: 19 (15–24) (median, Q1, Q3). Furthermore, a multiple linear regression analysis indicated that cyclothymic (*β* = 2.1, *p* = 0.048) and depressive (*β* = 1.2, *p* = 0.001) temperament traits and an insecure attachment (ASQ-anxious: *β* = 0.19, *p* = 0.006; ASQ-avoidant: *β* = 0.07, *p* < 0.001) predicted pandemic-related distress.

**Conclusions:**

Dimensions of both affective temperaments and attachment styles were associated with stress in the medical students during the transition to the endemic phase. The investigations of these psychological variables provided new information regarding risk factors for endemic-related distress, and pointed to potential targets for counseling and developing programs to support the medical students' mental health.

## 1. Background

Since the World Health Organization (WHO) officially announced the Corona virus disease-19 (COVID-19) pandemic [1], the ongoing COVID-19 health crisis has caused a dramatic burden on people's mental health [2]. Several reports indicated that the pandemic caused an increase in the prevalence of depression, insomnia, anxiety, and distress in at least one-third of the general population [3]–[5]. Furthermore, the prevalence of mental health symptoms was higher in people with occupational exposure risks, such as healthcare workers [6]. For instance, a previous study evaluated the resilience levels of nurses during the COVID-19 pandemic. Resilience was higher during the initial phase, but depression increased later. Self-efficacy, optimism, and emotional intelligence were found to be significant predictors of resilience, and emphasized the importance of fostering these factors to enhance healthcare workers' resilience and prevent burnout [7]. Moreover, while data may not always be consistent across studies, findings from the early stages of the pandemic suggest that females in the general population were more susceptible to experiencing anxiety [8]. In addition, the measures implemented to contain the pandemic and quarantine substantially impacted mental health due to frustration, boredom, isolation, and changes in personal social interactions [9]–[11].

The medical curriculum is typically very demanding and challenges medical trainees' mental health [12]. Medical students are subject to stressors that are typical nearly all college students: financial hardship, health risk behaviors, sleep deprivation, peer pressure, and extreme competition [13]. Although previous studies extensively assessed medical students' mental health during the pandemic [14], little is known about the endemic-related stress in medical students. During the endemic phase of COVID-19, stress levels in healthcare and education sectors remained significantly high and medical students were required to change their academic and clinical settings and their attitude toward patient' care [15]. Specifically, the aftermath of the pandemic posed challenges for medical students who experienced stress in transitioning to the conventional rhythms of academic and clinical activities [16]. The disruptive impact of the pandemic on medical education, as characterized by canceled rotations, postponed examinations, and the transition to remote learning modalities, added to the complexity of adjustments to in-person education [17]. Furthermore, the post-pandemic landscape introduced uncertainties in career trajectories, thereby intensifying the burden of expectation management [18]. Thus, even as the pandemic ended, the residual effects of these stressors persisted, thus perpetuating a cycle of psychological distress [19]. Understanding the role of emotional reactivity and attachment during this new phase is essential to provide targeted support and interventions to promote the students' mental health and resilience. According to Akiskal's model, five temperament traits describe the emotional reactivity types that characterize healthy subjects' behavioral patterns and individuals with an affective disorder spectrum [20]. These traits are stable, innate, and predict psychopathology [21]. Previous studies that used the Temperament Evaluation Memphis, Pisa, Paris, and San Diego Auto-questionnaire (TEMPS-A) described an association of temperament traits with psychological distress during the pandemic. Furthermore, affective temperament traits can predict the perceived stress, anxiety, depression, and health-risk behaviors in medical students [22]–[25]. They have been employed to investigate the effect of the COVID-19 outbreak on mental health in both the general population and in psychiatric patients [26]–[29].

Previous studies have shown that attachment relationships are essential when coping with stressful events [30]. The attachment theory suggests that children develop an emotional and behavioral substrate that regulates interactions and closeness with caregivers [31]. These interactions can grow in a positive way, thus resulting in a secure attachment. Conversely, if caregivers do not provide sensitive and meaningful interactions, children may develop an insecure attachment that indicates difficulty and distrust toward protective figures [31]. According to this theory, the attachment we develop during childhood affects our romantic and social interactions in adulthood and regulates our emotional reactivity [32]. For instance, adult attachment styles have been categorized as secure, anxious, and avoidant [33]. Individuals with a secure attachment rely on social support (a person's social networks and romantic relationships) to reduce arousal, emotional reactivity, and anxiety triggered by stressful events [34],[35]. On the contrary, individuals with insecure, avoidant adult attachment styles are uncomfortable with closeness and rely on themselves when facing stressful events. An insecure, anxious attachment style leads to reassurance-seeking and a dependence on partner support [36],[37]. The Attachment Style Questionnaire (ASQ) [38],[39] probes this theoretical framework. Previous studies in college students and the general population [40],[41] support the hypothesis that an insecure adult attachment is associated with stress and psychological morbidities during the COVID-19 pandemic.

In light of the aforementioned literature, this study aims to investigate medical students' distress at a major medical university in China during the transition to the endemic phase of COVID-19. We hypothesized that specific temperament traits and insecure attachments might be particularly relevant in explaining medical students' distress during the transition to the endemic phase.

## 2. Materials and methods

### 2.1. Design

A cross-sectional study was conducted at the Southern Medical University, Guangdong, China. All participants provided written informed consent before beginning the surveys. This study followed the Strengthening the Reporting of Observational Studies in Epidemiology (STROBE) reporting guidelines for cross-sectional studies. The study was approved by the ethics committee of the Southern Medical University, Guangdong, China (protocol number: 202207650) and was conducted following the principles of the Declaration of Helsinki. Informed consent was obtained from all subjects involved in this study.

This cross-sectional, online study was conducted in May 2022. The eligible individuals included all medical students (*N* = 567) enrolled at the Southern Medical University who could read and sign the informed consent section. Data were collected using the online software WENJUANXING (www.wjx.cn.). The first page of the electronic questionnaire included a description and purpose of the study, statements regarding confidentiality, and the voluntary basis of participation in the study. Students that submitted the questionnaire were considered to have provided their informed consent. Participants with diagnoses of psychiatric diseases were prevented from progressing with the questionnaire. All of the data were collected anonymously, the participation was voluntary, and the students did not receive compensation.

The survey included sociodemographic questions (e.g., age, gender, marital status, and academic year) and standardized questionnaires that investigated pandemic-related distress, affective temperament traits, and adult attachment styles in relationships.

### 2.2. Instruments

The Kessler Psychological Distress Scale (K10) is a self-rated 10-item questionnaire intended to investigate the distress that a person has experienced in the most recent 30 days [42]. The Chinese version of the scale was previously validated [43]. Each of the items is rated with a 5-level frequency Likert scale: 1. None of the time; 2. A little of the time; 3. Some of the time; 4. Most of the time; and 5. All of the time. The sum of each of the ten questions yields a score ranging from 10 to 50, with higher scores pointing to a more significant mental distress [42]. In this study, the Cronbach's alpha value of the K10 scale was 0.89.

The Temperament Evaluation of Memphis, Pisa, Paris, and San Diego-auto-questionnaire short version (TEMPS-A) is a self-administered 39-item, true-false questionnaire measuring five dimensions of affective temperament (including cyclothymic, 12 items; depressive, irritable, hyperthymic, 8 items each; and anxious, 3 items). The score is obtained by summing the items after dividing them by subscales (false = 1; true = 2) [14]. The Chinese version of the scale was previously validated [44]. In this study, the Cronbach's alpha values of the TEMPS-A short version ranged from 0.71 to 0.84.

The Attachment Style Questionnaire (ASQ) is a self-administered questionnaire consisting of 40 items to be answered using a 6-point Likert scale ( 1 = “totally disagree” and 6 = “totally agree”) [38]. The Chinese version of the scale was previously validated [45]. The subdomains include “Confidence”, “Discomfort with closeness”, “Relationships as secondary”, “Need for approval”, and “Preoccupation with relationships”. According to the attachment theory [34], confidence describes a secure attachment (ASQ-Secure), a discomfort with closeness and relationships as a secondary assess attachment avoidance (ASQ-Avoidance), and a need for approval and preoccupation with relationships assess attachment anxiety (ASQ-Anxiety). In this study, the Cronbach's alpha value of the secure, avoidant, and anxious attachment styles ranged from 0.68 to 0.75.

### 2.3. Statistical analysis

Descriptive statistics of the demographic variables, the students' distress, the affective temperament dimensions, and the adult attachment styles were generated. The IBM Statistics software, version 25 (IBM Corp., Armonk, NY, USA), was used to perform the statistical analyses. First, we investigated if the data were normally distributed using the Kolmogorov- Smirnov normality test. Then, we performed a correlation analysis of the continuous variables using the Spearman's correlation coefficient. The variables were subsequently entered into a multiple linear regression analysis model to investigate the predictors of the students “distress” during the aftermath of COVID-19. We assessed multicollinearity using the variance inflation factor (VIF). The alpha level was set at 0.05, and a *p*  < 0 .05 was statistically significant.

## 3. Results

### 3.1. Participants characteristics

We conducted an a priori power analysis using G*Power, version 3.1.9.7 [46], to determine the minimum sample size required to test the study hypothesis. The results indicated that the required sample size to achieve 80% power to detect a medium effect was *N* = 232 for a multiple linear regression analysis, at a significance criterion of *α* = 0.05. Thus, the obtained sample size of *N* = 402 is adequate to test the study hypothesis. The mean participant age was (21.3 ± 3.1). Most of the participants were female (*N * =  251, 62.4%), and the majority were single (*N*  = 331, 80%). 119 (29.6%) were freshman (Table 1).

**Table 1.** Demographic characteristics of the study population (*N* = 402).

***Project***	Data
***Age (Mean ± SD)***	21.3 ± 3.1
***Gender***
Male	151	37.6%
Female	251	62.4%
***Relationship***
Single	321	80%
Partnered	81	20%
***Academic Year***
Year 1	119	29.6%
Year 2	58	14.4%
Year 3	32	8%
Year 4	16	4%
Year 5	110	27.4%
Year 6	22	5.5%
Year 7	21	5.2%
Year 8	24	6%

All the data analyzed in the study (temperament and attachment style dimensions) did not meet the criterion of compliance with the normal distribution. Hence, the data are reported as median and Q1 and Q3 quartile (Table 2).

**Table 2.** Psychological variables. Data presented as median (Interquartile range, Q1–Q3) (*N* = 402).

**Temperament Traits**	Data
TEMPS-A Cyclothymic	0.6 IQR (0.3–1)
TEMPS-A Depressive	0.3 IQR (0–1)
TEMPS-A Irritable	0.1 IQR (0–1)
TEMPS-A Hyperthymic	0.5 IQR (0.1–1)
TEMPS-A Anxious	0.7 IQR (0.3–1)
**Adult attachment styles**
ASQ-Confidence	32 IQR (28–37)
ASQ-Anxious	28.3 IQR (24–31)
ASQ-Avoidant	24.5 IQR (21–28)
Stress	19 IQR (15–24)

Note: TEMPS-A, Temperament Evaluation of Memphis, Pisa, San Diego-auto questionnaire; ASQ: Attachment Styles Questionnaire. ASQ-Avoidant was obtained averaging the “Discomfort with Closeness” and “Relation as Secondary” dimensions; ASQ-Anxious was obtained by averaging the “Need for approval” and the “Preoccupation with Relationships dimensions”.

Furthermore, the correlation analysis showed that the distress was positively correlated with the cyclothymic (*r* = 0.173, *p* < 0.01) and depressive (*r* = 0.198, *p* < 0.01) TEMPS-A subscales scores. The secure attachment style (ASQ-Confidence) was inversely correlated with stress (*r* = −0.099, *p* < 0.05), while the insecure attachment styles scores were associated with an increased stress (ASQ-Anxious: *r* = 0.418, *p* < 0.01; ASQ-avoidant: *r*= 0.547, *p* < 0.01) (Table 3).

We carried out a multiple regression analysis to determine the variables that better predict stress in these medical students. The coefficient of determination (*R^2^* = 0.36) indicates that the regression equation predicted 36% of the variance, further suggesting that the model has a good prediction power for the dependent variable. The ANOVA F-value (*F* = 18.36, *p* < 0.0001) indicates a significant and linear relationship between the predictor criterion variables. The results indicate that both the cyclothymic (*β* = 2.1, *p* = 0.048) and depressive (*β* = 1.2, *p* = 0.001) temperament traits are positive predictors of stress. Furthermore, insecure attachment styles can predict the criterion variable (ASQ-anxious: *β* = 0.19, *p* = 0.006; ASQ-avoidant: *β* = 0.07, *p* < 0.001) (Table 4). The VIF was <2. 1 for all the predictor variables, excluding a significant multicollinearity.

**Table 3.** Associations between stress with temperaments traits score and adult attachment styles score, and age (*N* = 402).

No	Project	1	2	3	4	5	6	7	8	9	10
1	Age	1	-	-	-	-	-	-	-	-	-
2	TEMPS-A cyclothymic	0.725**	1	-	-	-	-	-	-	-	-
3	TEMPS-A depressive	0.646**	0.680**	1	-	-	-	-	-	-	-
4	TEMPS-A irritable	0.637**	0.591**	0.566**	1	-	-	-	-	-	-
5	TEMPS-A hyperthymic	0.747**	0.771**	0.680**	0.638**	1	-	-	-	-	-
6	TEMPS-A anxious	0.709**	0.759**	0.683**	0.591**	0.786**	1	-	-	-	-
7	ASQ-confidence	−0.441**	−0.454**	−0.568**	−0.421**	−0.519**	−0.601**	1	-	-	-
8	ASQ-anxious	0.001	0.110*	0.068	0	−0.101*	0	−0.062	1	-	-
9	ASQ-avoidant	−0.051	0.084	0.053	−0.084	−0.168**	−0.003	0.019	0.581**	1	-
10	Stress	0.054	0.173**	0.198**	−0.017	−0.026	0.084	−0.099**	0.418**	0.547**	1

Note: * *p*<0.05, ** *p*<0.01. TEMPS-A, Temperament Evaluation of Memphis, Pisa, San Diego-auto questionnaire; ASQ: Attachment Styles Questionnaire.

**Table 4.** Multiple linear regression model for predictors of medical students', Dependent variable: students' stress (*N* = 402).

**Project**	***B***	***Std. Error***	***p***	**95.0% *CI***	
				Lower Bound	Upper Bound
(Constant)	0.275	3.912	0.944	−7.416	7.966
Sex	0.038	0.611	0.95	−1.163	1.24
Age	0.001	0.156	0.996	−0.305	0.307
Relationship status	1.228	0.75	0.102	−0.247	2.704
Academic year	−0.027	0.134	0.841	−0.29	0.236
TEMPS-A Cyclothymic	2.107	1.061	0.048*	0.02	4.193
TEMPS-A Depressive	1.201	0.367	0.001**	0.481	1.922
TEMPS-A Irritable	−1.01	0.54	0.062	−2.071	0.051
TEMPS-A Hyperthymic	−1.517	0.916	0.098	−3.318	0.283
TEMPS-A Anxious	−0.374	0.926	0.687	−2.194	1.446
ASQ-confidence	−0.02	0.034	0.554	−0.086	0.046
ASQ-anxious	0.19	0.068	0.006**	0.055	0.324
ASQ-avoidant	0.544	0.07	<0.0001**	0.406	0.683

Note: *p < 0.05, **p < 0.01.

## 4. Discussion

Transitioning to the endemic phase of COVID-19 can be challenging and stressful for medical students. Investigating factors related to stress during this period is paramount. In a multiple regression analysis, the cyclothymic and depressive temperament traits were significant predictors of distress. Furthermore, the anxious and avoidant attachment styles in relationships were associated with higher stress levels in the medical students.

We report an association between cyclothymic and depressive temperament scores and stress. Our results coincide with the previously published literature. For instance, the cyclothymic temperament is characterized by cyclical mood swings, with periods of hypomania and depression [20]. These features have similarities to bipolar disorder, though they are “subthreshold” and do not meet the criteria for a diagnosis of bipolar disorder [47]. Yet, these traits influence behaviors and emotional reactivities and impact daily activities [48]. Individuals with high scores on the cyclothymic subscale demonstrated symptoms of anxiety and depression and were more likely to develop mood disorders and even bipolar disorder later in life [49]. Furthermore, our findings align with the available literature, which indicates that cyclothymic temperament can make a person more vulnerable to stress [20]. The rapid mood changes characteristic of this trait interfered with the coping strategies that were implemented to deal with daily stressors [22]. People with cyclothymic temperament may also have more difficulty regulating their emotions and managing stress in general [20]. Similarly, depressive temperament traits are characterized by excessive self-esteem, pessimism, rumination, and apathy [20]. Individuals with a depressive temperament are more empathic and prone to guilt. High scores in depressive traits have been associated with stress, burnout, and mood disorders [50].

Our data confirmed and expanded the findings of other studies that investigated the association of temperament traits with a positive mental status in medical students during the COVID-19 pandemic. Cyclothymic and depressive traits inversely correlated with mental flourishing in a cohort of Italian medical students [51] and predicted health-risk behaviors and perceived stress in medical students [24],[25]. Our study validated the impact of cyclothymic and depressive traits on mental health in different cultural and pandemic settings. Taken together, these results support the use of TEMPS-A to investigate the harmful effects of the pandemic on medical students' mental health. Moreover, studies in the general population [26] and healthcare workers [52] further support our findings.

We showed that the secure adult attachment style inversely correlated with stress. In contrast, higher scores in the insecure attachment dimensions (anxious and avoidant) were associated with and predicted stress in Chinese medical students during the time of our survey. Previous research indicates that individuals with habitual and rewarding loving relationships demonstrated excellent social interactions, high mental health, and low psychiatric morbidity [53],[54]. Our results are consistent with this view and further support the attachment theory conceptualization of a relationship as an emotional aid in response to stressful and adverse events [55]. The data were consistent with previous results that indicated a link between secure attachment adaptative coping, empathy, and resilience [56]. In contrast, individuals with an insecure-anxious attachment style were self-doubting and depended on others for validation. At the same time, they displayed proximity-seeking behaviors, a fear of rejection, and a distrust of others. Medical students with high scores in the insecure-avoidant dimension were afraid of intimacy and lacked empathy [57]. Additionally, they had a negative view of themselves and were overly independent [39]. Our results support the notion that individuals with insecure attachments have difficulty managing stressful events. For instance, individuals with an insecure-anxious attachment style have difficulty trusting others, making it harder for them to reach out for help when needed [35]. Additionally, they may experience more intense emotional reactions to stressors. Individuals with insecure-avoidant attachment style may have difficulty forming close relationships, which may result in them not having a support system to rely on during times of stress [35],[36].

The current study conceptual frameworks are rooted in stress theories that explain how an individual's response to stressors can affect their physical and mental health. According to Lazarus and Folkman [58], a medical students' stress may develop through abnormal cognitive appraisal and coping. To this extent, temperament traits and the consequent excessive emotional reactivity coupled with situational factors (fear of infection, curricular and social/behavioral changes) may lead to maladaptive emotional coping. Thus, stress may occur when the students perceive a situation as threatening and feel they do not have the resources to cope [51]. The allostatic load theory was described by McEwen and Stellar in 1993 [59] and focused on the physiological effects of chronic stress. According to this theory, allostatic load refers to the bodily and mental adaptations (hormonal, immunologic, inflammatory, trophic, and plastic) to chronic environmental stressors. Stress occurs when an individual perceives a new situation as threatening and feel that they do not have the resources to cope with it. This perception triggers the body's “fight or flight” response, which leads to maladaptive neuronal plasticity and, ultimately, a predisposition to anxiety and depression [59],[60]. Medical students are regularly exposed to academic stress and display abnormal cortical plasticity and metaplasticity [61]–[63]. Our data suggests that the students' innate emotional reactivity and attachment style may alter their coping strategies, which increases the allostatic load of life events. To this extent, the cognitive load theory [64] states that our cognitive capacity is limited, and excessive (cognitive) demands can result in an overload and decreased performance. The pandemic fatigue resulting from multiple pandemic waves can also be considered a specific and severe form of cognitive load as defined under the cognitive load theory [65]. It may be relevant to understand stress in students enrolled in a high demanding medical curriculum. Lastly, previous studies highlighted the interplay between stressors and relationship quality among the general population [66],[67]. According to the socioemotional selectivity theory (SST) [68], the impact of pandemic-related stress may be particularly pronounced for individuals who place a high value on emotional closeness. SST can be used to understand the pandemic's effects on the medical students' social and emotional goals and how these may have changed because of the pandemic. As previously reported during the SARS epidemic in Hong Kong, individuals prioritize emotionally close relationships during a pandemic. The pandemic restrictions interfere with social and emotional goals and regulation strategies during this time. They may be pivotal for the development of stress, anxiety, fear, sadness, and loneliness, regardless of the individual's age [69],[50]. While the transition to the endemic phase has enabled the partial restoration of social support networks, medical students are still experiencing significant stressors related to COVID-19 and the adjustments required in their academic and clinical environments. Institutions need to adapt their support strategies to effectively address these evolving needs [70]. Overall, our results are consistent with relevant frameworks and highlight the importance of innate determinants of emotional reactivity and secure attachments when medical students face unprecedented, repeated threats.

This study has some limitations. First, the cross-sectional design makes it impossible to infer causality. Since our study included medical students from one medical school, we cannot rule out a selection bias, thus limiting the results' generalizability. In addition, data were collected through self-reporting, which may be biased because individuals tend to report more socially acceptable answers, and the rehearsal of negative autobiographical memories may influence the results [71]. Furthermore, future research should aim to incorporate a more comprehensive demographic evaluation to enhance the generalizability of the findings. Despite these limitations, the study was well-powered and was the first reported study to address the association between these variables in a population of Chinese medical students during the aftermath of the COVID-19 Omicron wave.

## 5. Conclusions

Our study highlights the role of temperament traits and healthy relationships that affect the mental well-being of medical students, especially during times of crisis and the transition to the new normal. Other contributing factors may include coping mechanisms, access to counseling, and stress-management services. Additionally, as attachment styles can change over time, providing appropriate mental health services based on our findings could help improve the mental health of medical students.

## Use of AI tools declaration

The authors declare they have not used Artificial Intelligence (AI) tools in the creation of this article.
